# Tissue engineering of heart valves: PEGylation of decellularized porcine aortic valve as a scaffold for *in vitro* recellularization

**DOI:** 10.1186/1475-925X-12-87

**Published:** 2013-09-05

**Authors:** Jianliang Zhou, Shidong Hu, Jingli Ding, Jianjun Xu, Jiawei Shi, Nianguo Dong

**Affiliations:** 1 Department of Cardiothoracic Surgery, the Second Affiliated Hospital of Nanchang University, Nanchang 330006, China; 2 Medical College of Nanchang University, Nanchang 330006, China; 3 Department of Cardiovascular Surgery, the Union Hospital Affiliated to Tongji Medical College of Huazhong University of Science and Technology, Wuhan 430022, China

**Keywords:** Decellularized valves, Polyethylene glycol, GRGDSPC peptides, VEGF165, Michael addition reaction

## Abstract

**Background:**

Poly (ethylene glycol) (PEG) has attracted broad interest for tissue engineering applications. The aim of this study was to synthesize 4-arm -PEG-20kDa with the terminal group of diacrylate (4-arm-PEG-DA) and evaluate its dual functionality for decellularized porcine aortic valve (DAV) based on its mechanical and biological properties.

**Methods:**

4-arm-PEG-DA was synthesized by graft copolymerization of linear PEG 20,000 monomers, and characterized by IR^1^H NMR and ^13^C NMR; PEGylation of DAV was achieved by the Michael addition reaction between propylene acyl and thiol, its effect was tested by uniaxial planar tensile testing, hematoxylin and eosin (HE) and scanning electron microscopy (SEM). Gly-Arg-Gly-Asp-Ser-Pro-Cys (GRGDSPC) peptides and vascular endothelial growth factor-165 (VEGF_165_) were conjugated onto DAV by branched PEG-DA (GRGDSPC-PEG-DAV-PEG-VEGF_165_).

**Results:**

Mechanical testing confirmed that PEG-cross-linking significantly enhanced the tensile strength of DAV. Immunofluoresce confirmed the GRGDSPC peptides and VEGF_165_ were conjugated effectively onto DAV; the quantification of conjunction was completed roughly using spectrophotometry and ELISA. The human umbilical vein endothelial cells (HUVECs) grew and spread well on the GRGDSPC-PEG-DAV-PEG-VEGF_165_.

**Conclusions:**

Therefore, PEGylation of DAV not only can improve the tensile strength of DAV, and can also mediate the conjugation of bioactive molecule (VEGF_165_ and GRGDSPC peptides) on DAV, which might be suitable for further development of tissue engineered heart valve.

## Introduction

Heart valve disease is considered to be among the many factors that are significant for mortality throughout the world. Prosthetic valve implantation utilizing a mechanical prosthetic valve or bioprosthetic heart valve is the most common treatment for valvular heart disease. However, both these approaches have shortcomings. These shortcomings including the risk of: embolism, wear and an inability to grow, which makes them unsuitable for treating congenital heart defects. Consequently, many researchers are now exploring tissue engineering strategies to develop heart valves equivalent [1,2].

Scaffold material is a key factor in preparing heart valves through tissue engineering.

Two categories of materials are used as the scaffold to construct TEHV, namely polymer scaffolds and decellularized allogenic or xenogenic scaffolds [3]. Polymer scaffolds can be derived from biological or synthetic source. Biological polymers possess intrinsic cell compatibility as most are structural components familiar to cells, but also tend to be mechanically weak and difficult to manipulate. Synthetic sources are easily tailored and can be reproduced readily, allowing for novel structural designs that can control mechanical properties, surface topography, and porosity with limitations including uncertainties regarding the degradation rate and products as well as cell compatibility [4,5]. Decellularized allogenic and xenogenic scaffolds have natural three-dimensional (3-D) structures and extracellular matrix (ECM) composition. However, because the application of human decellularized allografts is limited due to worldwide organ scarcity, decellularized xenogeneic tissues become highly attractive scaffold materials for tissue engineering (TE) [6]. Among heterogeneous decellularized valves, especially decellularized porcine aortic valves have been used for the preparation of tissue engineered heart valves (TEHV) [7-9], due to their similarity with human heart valves in 3-D structures, ECM composition, and rich source [6,10,11]. However, studies found that decellularization may lead to part of the collagen fibers may breaking and a reduction of collagen cross-linking, which in turn may decrease the mechanical strength including ultimate tensile strengths and elastic modulus of the porcine aortic heart valve; in addition loss of extracellular matrix components may affect the adhesion and proliferation of seeded cells on the valve [12-16]. What’s more, adequate mechanical stimuli, which can be provided by the dynamic bioreactor in vitro, are conducive to improve the relevant mechanical properties of the valvular complex, such as the mechanical strength and stiffness [17]. Therefore, to overcome these limitations produced by decellularization, it is necessary to find a method or material with good mechanical properties and good biological activity.

Covalent attachment of synthetic polymers to biological macromolecules offers an effective means to modify their properties, providing enhanced functionality for drug delivery and tissue engineering. One of the common approaches to achieve this goal is the covalent attachment of poly (ethylene glycol) (PEG) to therapeutic proteins, termed PEGylation. Therefore, the objective of the present study was to improve the mechanical properties of decellularized porcine aortic valve (DAV) and to promote its endothelialization, which would further facilitate the development of TEHV. To achieve this objective, we aimed to synthesize branched 4-arm poly (ethylene glycol) with the terminal group of diacrylate (PEG-DA), DAV cross-linked by Michael addition reaction between acryloyl with thiol. We also aimed to covalently link the biological signaling molecules (RGD peptide and VEGF) to the decellularized porcine aortic valve.

## Materials and methods

### Materials

PEG 20000, tert-octylphenylpolyoxyethylen (Triton-100), ethylenediaminetetraacetic acid (EDTA), RNase A, DNase, and human recombinant VEGF_165_ were purchased from Sigma. N-succinimidyl-S-acetylthioacetate (SATA) was purchased from Pierce (Rockford, IL, USA). Gly-Arg-Gly-Asp-Ser-Pro-Cys (GRGDSPC) peptides and FITC- Gly-Arg-Gly-Asp-Ser-Pro-Cys (FITC-GRGDSPC) peptides were obtained from GL Biochem (Shanghai) Ltd. Water used in this study was distilled twice. This study was approved by the ethics committee of the Second Affiliated Hospital of Nanchang University, China.

### Synthesis and characterization of branched 4-arm-PEG-DA

**Figure 1:**
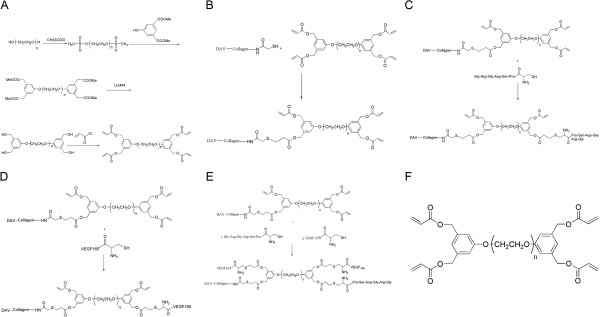
**Schematic representation of the branched 4-arm PEG-DA and different reactions. A**: Schematic representation of the synthesis of branched 4-arm PEG-DA. **B**: Schematic representation of the PEGylation of decellularized valves. **C**: Schematic representation for the immobilization of decellularized valves by the GRGDSPC peptide. **D**: Schematic representation for the immobilization of decellularized valves by the VEGF_165_. **E**: Schematic representation for the simultaneous immobilization of decellularized valves by GRGDSPC peptide and VEGF_165_. **F**: The structure of the branched 4-arm PEG-DA.

The branched PEG-DA used in this study was synthesized by us. The schematic representation of the synthesis is provided in Figure 1A. Briefly, linear PEG (MW = 20kDa) was dissolved in dichloromethane, and allowed to react with methanesulfonyl chloride, 5-hydroxyl-mono-methyl isophthalate, lithium aluminium tetrahydride, and acryloyl chloride, followed by purification procedures, to form branched PEG-DA. IR, ^1^H NMR and ^13^C NMR were used to verify the final product.

### PEGylation of decellularized valves

The schematic representation for the PEGylation of decellularized valves (*i.e.* construction of PEG-DAV or PEG-crosslinking of decellularized valve) is presented in Figure 1B.

Porcine hearts were obtained from a local slaughterhouse. The porcine aortic valves were excised from the hearts under sterile conditions; decellularization was performed as described previously [18-20]. However, in this study, several modifications were applied. Briefly, after removal of adherent tissue the aortic valve leaflets were placed in a solution of 0.05% Triton-100 with 0.02% EDTA in phosphate buffer solution (PBS) without Ca^2+^ and Mg^2+^ for 24 hours under continuous shaking at 37°C, together with RNase A (0.02 mg/mL) and DNase (0.2 mg/mL) followed by PBS supplemented with penicillin and streptomycin flushing for 48 h with constant shaking to remove all cellular debris. The valves were then stored in PBS at 4°C before further processing and seeding. To characterize the decellularized scaffolds, routine histological examination by hematoxylin and eosin (HE) and scanning electron microscopy (SEM) were performed [1,6,13,21].

SH-groups (Thiols) were introduced into the decellularized valve leaflets by a method similar to the one previously described [22]. Briefly, forty decellularized valve leaflets (mean dry weight 6.47 ± 1.34 mg per sample) were immersed in 50 mL of 50 mM PBS (pH7.6, containing 1 mM EDTA) and allowed to react with 50 mg SATA for 2 h at room temperature under continuous shaking. The reaction was then stopped by extensive washing procedure with PBS. The acetylated sulfhydryl groups were deprotected by adding 50 mL of 0.5 M hydroxylamine hydrochloride (NH_2_OH · HCl) in 50 mM PBS (pH7.6) for 2 h at room temperature. Subsequently the leaflets were extensively washed with PBS to remove the redundant hydroxylamine hydrochloride. To test the extent of SH-modification, the leaflets which had been treated with SATA and hydroxylamine hydrochloride were allowed to react with 10 mM DTNB (Ellman’s reagent, 5,5’-dithio-bis-[2-nitrobenzoicacid], Sigma) in 100 mL disodium hydrogen phosphate solution (50 mM, pH 8.0) for 15 min. After determination of the absorbance at 412 nm, the sulfhydryl group contents of the decellularized valve leaflets was deduced from standard curves obtained with cysteine [23]. Forty decellularized valve leaflets with introduced sulfhydryl groups were immersed in 50 mL 0.1 M PBS (pH7.5, 5 mM EDTA), and allowed to react with 500 mg branched 4-arm-PEG-DA for 8 h at 37°C under constant stirring. Finally, Samples were rinsed with PBS to remove the excess PEG-DA. Morphological analysis of PEGylated valves were performed using HE and SEM.

To evaluate the biomechanical properties of the PEGylated valves compared to native and decellularized valves, the leaflets specimens were subjected to uniaxial planar, tensile testing [13,24]. Specimens of 3 mm × 15 mm were cut out from the valve leaflets in the circumferential fiber direction. The tensile tests were performed on an Instron 5848 MicroTester (Instron Corporation, U.S.A.) at a constant speed of 5 mm/min until specimen fracture. Six specimens were tested in each group [6].

### Preparation of GRGDSPC-PEG-DAV-PEG-VEGF_165_

The GRGDSPC peptide and Human recombinant VEGF_165_ was seperately immobilized covalently on decellularized valves by PEG-DA to find respective optimum reaction conditions (Schematic representations are provided in Figure 1(C,D)). The PEG-crosslinked decellularized valve was each reacted with 1 mL GRGDSPC peptide and Human recombinant VEGF_165_ solution of different concentrations at 37°C. The amount of conjugation was evaluated quantitatively by spectrophotometry and ELISA at different time points, and the qualitative tests were performed by immunofluorescence (FITC-Ahx- Gly-Arg-Gly-Asp-Ser-Pro-Cys, FITC-GRGDSPC,rabbit anti-human vascuoar endothelial cell growth factor and goat anti-rabbit IgG/Cy3). The decellularized valves were used as a negative control.

Considering respective optimum reaction conditions, The PEG-crosslinked decellularized valves were reacted with 1 mL reaction mixture solution (including 1 mg/mL GRGDSPC peptide and 1000 pg/mL VEGF_165_) at 37°C for 4 h. Then the immunofluorescence was analyzed for qualitative evaluation of the conjugation effects. The decellularized valves were used as a negative control (A schematic representation is provided in Figure 1E).

### Biological function of signal molecules immobilized covalently on decellularized valves

After being cut into equal size disks (diameter = 1cm), DAVs, PEGylated decellularized aortic valves (PEG-DAV) and RGD and VEGF-conjugated PEGylated decellularized aortic valves (GRGDSPC-PEG-DAV-PEG-VEGF_165_) were put into different wells in a 24-well culture plate. HUVECs (KG110, Nanjing KeyGEN, China) [25] at 80% confluence were harvested using a 0.05% trypsin solution and condensed to a concentration of 1 × 10^6^/mL with serum-free media. A cell suspension of 0.5 mL was dropped on the top of the scaffolds and incubated in humidified air with 5% CO_2_ at 37°C for 2 h to allow adherence of the cells onto the scaffolds. Following this incubation, unattached cells were removed with PBS, the cells/scaffolds constructs were then transferred into another 24-well plate and cultured for the desired time period.

The DNA content of the scaffold seeded HUVECs was quantified with Hoechst Dye 33258 (Invitrogen, USA) at 4 and 8 days. The cells were harvested from six samples of each group with 0.05% trypsin. Cells were centrifuged, lysed and then diluted lysates were incubated with an equal volume of 0.1 mg/mL Hoechst 33258 Dye for 5 min in 96-well plates protected from light. Fluorescence was determined with a FLUOstar Optima fluorescent plate reader (BMG Labtech, Offenburg, Germany) at 352 nm excitation and 461 nm emission. The DNA content in each sample was determined according to a DNA standard curve. SEM was performed for each group after 8 days of culture.

### Statistical analysis

All quantitative data were expressed as mean ± standard deviations (mean ± SD). One-way analysis of variance (ANOVA) was performed and confidence intervals (CI) were determined using a statistical program (SPSS, Version 13.0, USA). *p* values of less than 0.05 were considered statistically significant.

## Results

### Identification of branched PEG-DA

The results of IR, ^1^H NMR and ^13^C NMR were as follows: IR (NaCl): 2883 cm^-1^, 1723 cm^-1^, 1467 cm^-1^, 1280 cm^-1^, 1112 cm^-1^, 946 cm^-1^, 842 cm^-1^; ^1^H NMR (CDCl_3_): 3.51-3.86 (OCH_2_CH_2_)_n_, 4.14 (4H, CH_2_OAr), 5.16 (8H, CH_2_-Ar), 5.88 (4H, CH=), 6.16 (4H, CH_2_=), 6.44 (4H, CH_2_=), 6.90-6.96 (6H, Ar); ^13^C NMR (CDCl_3_): 166.2, 159.3, 137.5, 131.2, 128.0, 113.9, 70.5. The structure of branched PEG-DA is given in Figure 1F.

### Characteristics of decellularization and assessment of sulfhydryl group introduction into the decellularized valves

Decellularization procedures removed all cellular components of the valve leaflets, whereas the extracellular matrix was well preserved and showed a loosened and porous structure. The amount of sulfhydryl introduced into the decellularized valve (per mg dry weight) was 0.815 ± 0.012 × 10^-7^mol by Ellman’s reagent.

### Scanning electron microscopy

**Figure 2:**
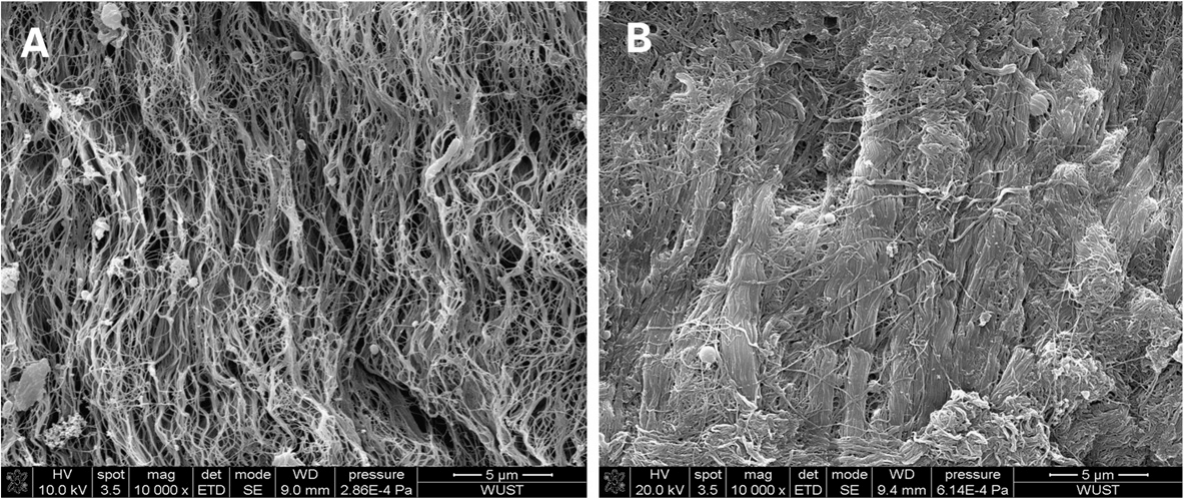
**Observations of decellularized valve and PEG-cross-linked decellularized valve under scanning electron microscopy.** SEM (×10000) of a decellularized valve **(A)** and a PEG-cross-linked decellularized valve **(B)**.

Compared to decellularized valves, the surface of PEG-cross-linked decellularized valves suggested they could form a thin film of PEG, the connections between the collagenous fibers were significantly increased and fiber bundles were thickened (Figure 2).

### Mechanical testing of various valve leaflets

**Figure 3:**
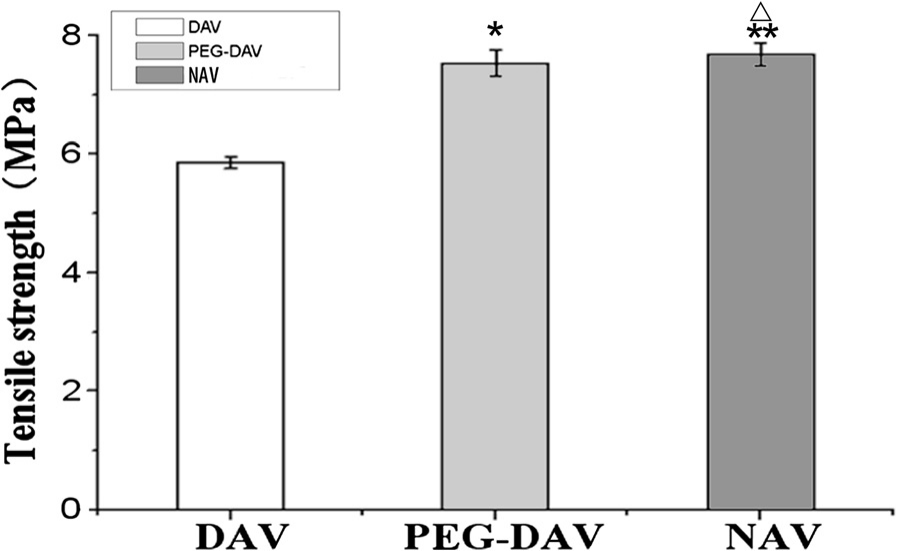
**The maximal tensile strength as measured by using Instron 5848 MicroTester (Instron Corporation, U.S.A.).** Decellularized aortic valves (DAV, 5.65 ± 0.24) *vs.* native aortic valves (NAV, 7.68 ± 0.19): *p* < 0.05; PEGylated decellularized aortic valves (PEG-DAV, 7.53 ± 0.25) *vs.* NAV: *p* > 0.05 n = 8.

It was found that the decellularization procedure reduced the maximal tensile strength of the acellular valves compared to the native valves (P < 0.05).There was no significant difference with respect to the mechanical tensile strength properties between PEGylated and native groups, n = 8 (Figure 3).

### Characteristics of GRGDSPC-PEG-DAV-PEG-VEGF_165_

**Figure 4:**
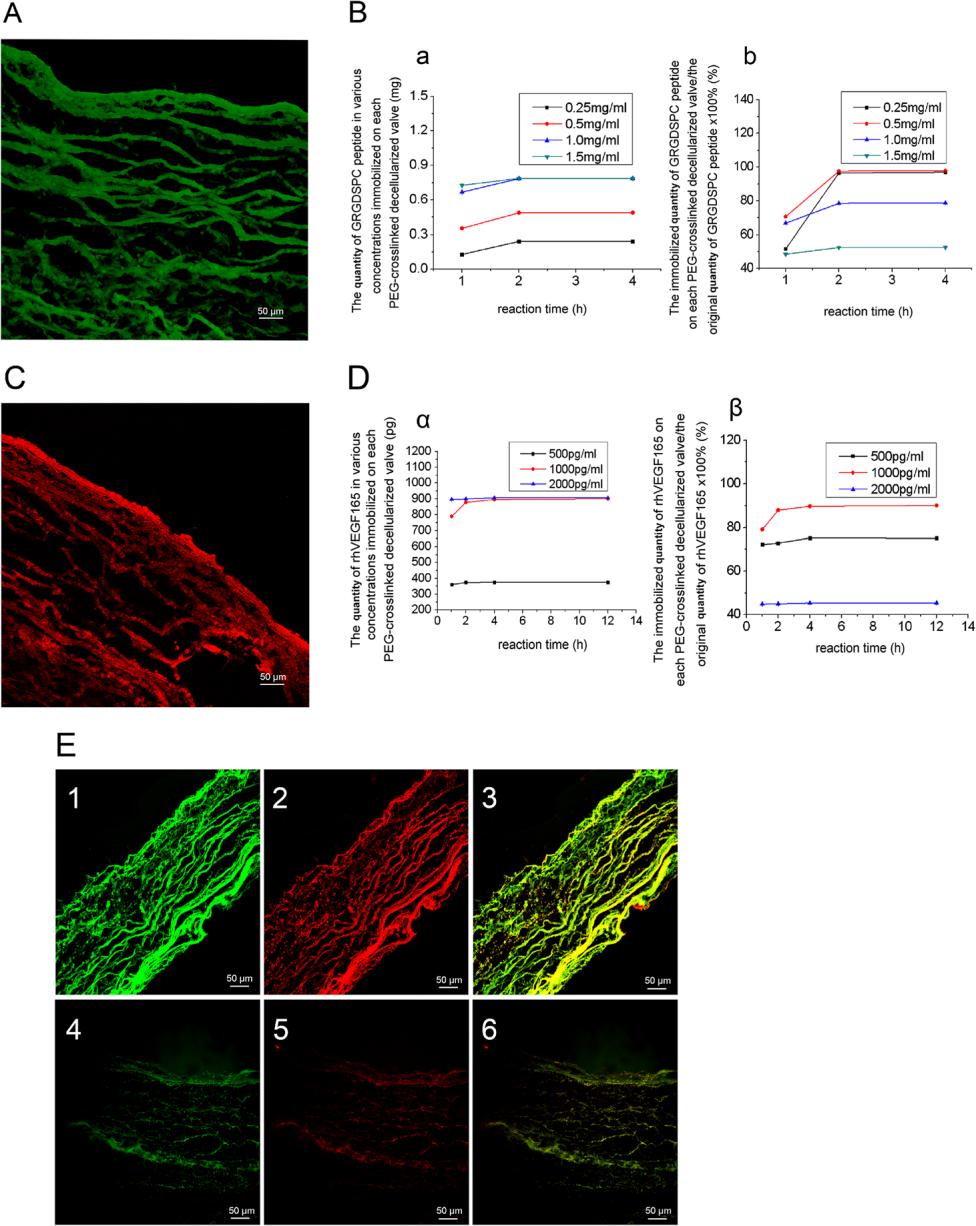
**The effect of conjugation under laser scanning confocal microscope and the amount of conjugation. A**: FITC-Ahx-GRGDSPC peptide conjugated on PEG-DAV under laser scanning confocal microscope (×200) Scale bars, 50 μm,meanwhile,the negative control appears few fluorescence. **B**: The quantity (mg) of GRGDSPC peptide in various concentrations immobilized on PEG-cross-linked decellularized valves at a different time points(a) and its proportion in the original GRGDSPC peptide (%)(b) **C**: VEGF_165_ conjugated on PEG-DAV under laser scanning confocal microscope (×200)Scale bars, 50 μm, meanwhile, the negative control appears few fluorescence **D**: The quantity (pg) of rh VEGF_165_ in various concentrations immobilized on PEG-cross-linked decellularized valve at different points(α) and its proportion in the original rh VEGF_165_ (%)(β) **E**: The express of GRGDSPC peptide (green) and VEGF_165_ (red) conjugated on DAV (4-6) and PEG-DAV (1-3) (×200) 1 and 4 are the express of GRGDSPC peptide, 2 and 5 are the express of VEGF_165_, 3 and 6 are the simultaneous express of GRGDSPC peptide and VEGF_165_. Scale bars, 50 μm.

The GRGDSPC peptides were conjugated effectively with the PEG-cross-linked decellularized valves, shown by the FITC-GRGDSPC peptide giving high fluorescence in the PEG-crosslinked decellularized valves. However, the negative controls showed little fluorescence (Figure 4A). Moreover, the quantity of conjugated GRGDSPC peptides reached a maximum amount of 0.79 ± 0.01 mg when the concentration of GRGDSPC peptides in solution was 1 mg/mL and the reaction time was 2 h (Figure 4B).

The human recombinant VEGF_165_ were conjugated effectively with the PEG-cross-linked decellularized valves, shown by the VEGF_165_ being highly positive in immunofluorescence in the PEG-cross-linked decellularized valves, but the negative controls showed little fluorescence (Figure 4C). Moreover, the quantity of conjugated VEGF_165_ reached a maximum of 896.87 ± 3.27 pg when the concentration of VEGF_165_ in solution was 1000 pg/mL and the reaction time was 4 h (Figure 4D).

The GRGDSPC peptides and human recombinant VEGF_165_ were conjugated effectively with the PEG-cross-linked decellularized valves simultaneously, since the GRGDSPC peptide and VEGF165 were highly positive in immunofluorescence of the PEG-cross-linked decellularized valves, but the negative controls showed little fluorescence (Figure 4E).

### Biological function of the GRGDSPC peptide and human recombinant VEGF_165_ immobilized covalently on decellularized valves: cellular immobilization and proliferation

**Figure 5:**
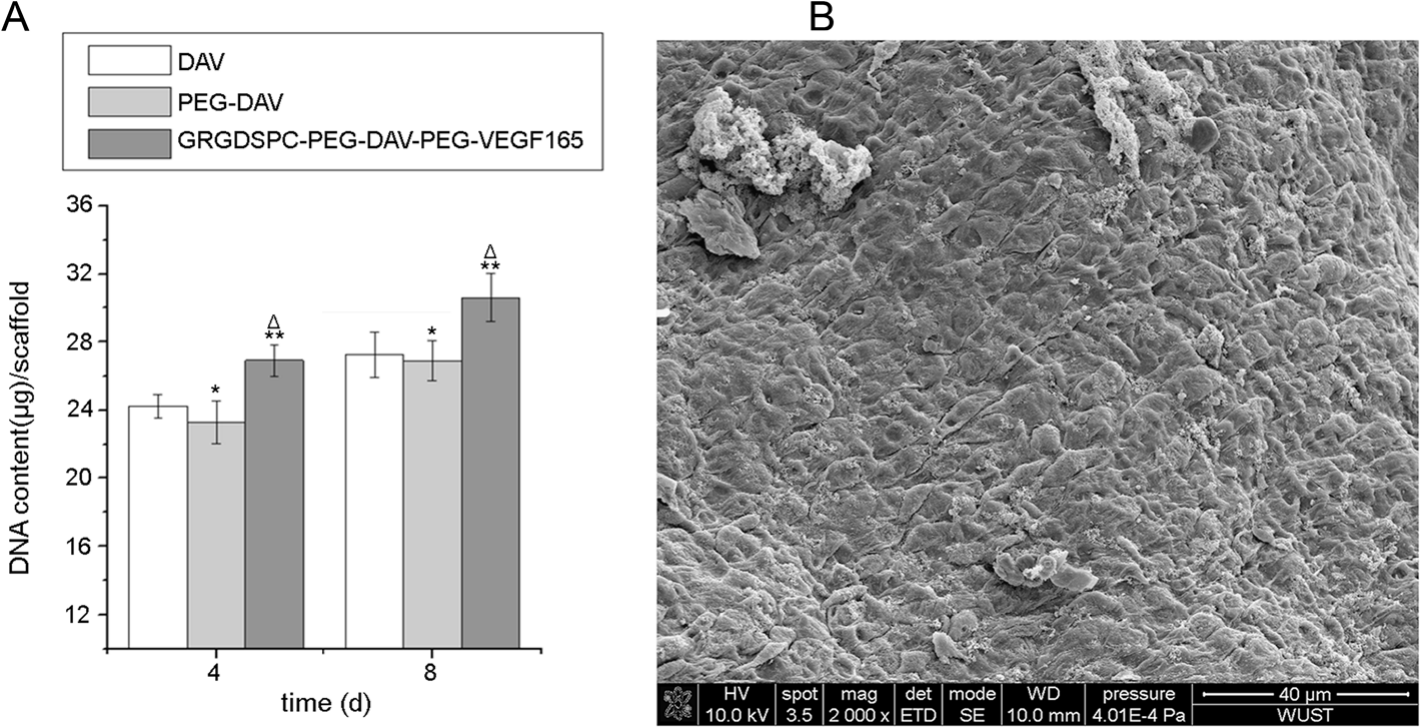
**The quantification of DNA at different time among groups and scanning electron photomicrographs of GRGDSPC-PEG-DAV-PEG-VEGF**_**165 **_**group. A**: Quantification of DNA in the DAV, PEG-DAV, GRGDSPC-PEG-DAV-PEG-VEGF_165_ groups at 4 and 8 days. Compared with DAV, *P > 0.05, **P < 0.05; compared with PEG-DAV, ΔP < 0.01 **B**: Scanning electron photomicrographs depicting HUVECs adhesion and proliferation on GRGDSPC-PEG-DAV-PEG-VEGF_165_ group after 8 days in culture (×2000).

The DNA content of the GRGDSPC-PEG-DAV-PEG-VEGF_165_ group at 4 and 8 days were 26.91 ± 0.88 μg/scaffold and 30.59 ± 1.42 μg/scaffold, respectively, while those in the DAV were 24.24 ± 0.68 μg/scaffold and 27.26 ± 1.35 μg/scaffold and in the PEG-DAV group were 23.28 ± 1.25 μg/scaffold and 26.9 ± 1.17 μg/scaffold. The DNA content of the DAV and PEG-DAV groups were significantly lower than those of the GRGDSPC-PEG-DAV-PEG-VEGF_165_ group at 4 and 8 days (Figure 5A). Meanwhile, SEM revealed that a confluent and compact monolayer was formed on the surface of the GRGDSPC-PEG-DAV-PEG-VEGF_165_ group, but the other groups had clusters of scattered cells with endothelial morphology (Figure 5B).

## Discussion

PEG is a hydrophilic polymer, which has a long flexible chain and is attractive to researchers into the polypeptide/protein drugs and molecular-targeted therapies because of its favorable biodegradability and biocompatibility [22,26,27]. Interestingly, functional groups [28] (such as vinyl sulphone, acryloyl) can be introduced into the terminal hydroxyl of PEG because of its strong controllability, which makes PEG widely used for chemical modification [29-34]. In the present study, the acroloyl-group was introduced into the end of every branch of PEG, which provided a method for the covalently binding of thiols.

PEGylation is the covalent attachment of PEG to biomolecules of interest, such as (poly) peptides and proteins. PEGylation was first reported by Abuchowski et.al in the 1970s for albumin and catalase modification [35,36]. Since then the procedure of PEGylation has been broadened and developed tremendously [37,38]. An important aspect of PEGylation is the incorporation of various PEG functional groups that are used to attach the PEG to the peptide or protein [39]. Collagen is one of the important components of the extracellular matrix of heart valves; decellularization retains most of the collagen component. Thus, PEGylation of decellularized valves is mainly PEGylation of collagen.

Crosslinking of decellularized valves required dual functionality of PEG-DA. For the first function, SH-groups were introduced into the collagen by SATA, then with excess of acroloyl-groups on PEG-DA compared with the SH-groups on the decellularized valves (the molar ratio of acroloyl-groups to SH-groups was 20:1), PEG-crosslinking of decellularized valves was accomplished by the Michael addition reaction of the acroloyl-groups and the SH-groups. In this study, the SEM showed that the surface of the PEG-cross-linked decellularized valves formed a thin film of PEG, the connection between collagenous fibers increased significantly and formed fiber bundles that were thicker than with the decellularized valves. The biomechanical tests showed that the tensile strength of the PEG-cross-linked decellularized valves had been notably enhanced compared with decellularized valves and there was no obvious difference with native valves. The insertion of PEG molecules among the collagen molecules might have cross-linked the collagen molecules to one another by the addition reaction of acroloyl-groups and SH-groups, which in turn made the space structure of collagen in the decellularized valves more stable.

The second function of PEG-DA allows the immobilization of biological signals, such as RGD peptides and VEGF, immobilized covalently into decellularized valves is another function of bifunctional PEG-DA. Using the Michael addition reaction of unsaturated acroloyl-groups in PEG-cross-linked decellularized valves (i.e. unreacted acroloyl-groups in PEG with SH-groups introduced into decellularized valves) with SH-groups in the GRGDSPC peptide or (and) VEGF, biological signal was immobilized covalently into decellularized valves. In this study, laser confocal microscopy observation and quantitative detection (spectrophotometry and ELISA) showed that GRGDSPC peptide and VEGF can not only be conjugated respectively, but can also be conjugated simultaneously by using PEG-DA. More importantly, these biological signals can not only be conjugated into decellularized valve, but also promote cell adhesion and cell proliferation, which was confirmed by the cellular DNA contents of each group and SEM.

## Conclusions

Using the Michael addition reaction of acroloyl-groups in PEG-DA and SH-groups, not only the PEG-cross-linking of decellularized valves can be accomplished, and biological signals can also be conjugated into decellularized valves, which can partially improve the partial mechanical and biological properties of decellularized valves. Therefore, the novel branched PEG-DA may be a potential cross-linking reagent for the modification of tissue engineering scaffolds, which is expected to break through the traditional glutaraldehyde cross-linking of bio-derived valve materials, and to overcoming the shortcomings of valve decellularization. Further research and exploration of GRGDSPC-PEG-DAV-PEG-VEGF_165_ should be made into their wear over time and fluid dynamic performance, as well as the interactions of signaling molecules cells and extracellular matrix *in vivo* or in a bioreactor.

## Abbreviations

PEG: Poly (ethylene glycol); DAV: Decellularized porcine aortic valve; HE: Hematoxylin and eosin; SEM: Scanning electron microscopy; GRGDSPC: Gly-Arg-Gly-Asp-Ser-Pro-Cys; VEGF165: Vascular endothelial growth factor-165; HUVECs: Human umbilical vein endothelial cells; TEHV: Tissue engineered heart valves; Triton-100: Tert-octylphenylpolyoxyethylen; EDTA: Ethylenediaminetetraacetic acid; SATA: N-succinimidyl-S-acetylthioacetate; FITC-GRGDSPC: Fluorescein isothiocyanate-Gly-Arg-Gly-Asp-Ser-Pro-Cys; PBS: Phosphate buffer solution; NH2OH · HCl: Hydroxylamine hydrochloride; DTNB: Ellman’s reagent/5,5’-dithio-bis-[2-nitrobenzoicacid]; PEG-DAV: PEGylated decellularized aortic valves; ELISA: Enzyme linked immunosorbent assay; 4-arm -PEG-DA: 4-arm -PEG-20kDa with the terminal group of diacrylate; H NMR: ^1^Hydrogen nuclear magnetic resonance (NMR) spectroscopy; C NMR: 13Carbon nuclear magnetic resonance (NMR) spectroscopy; 3-D: Three-dimensional; ECM: Extracellular matrix.

## Competing interests

The authors declare that they have no competing interests.

## Authors’ contributions

Each Author has contributed substantially to the research, preparation and production of the paper and approves of its submission to the Journal. All authors read and approved the final manuscript.

## Authors’ information

Jianliang Zhou and Shidong Hu are co-first authors.
